# Interoperability between Biomedical Ontologies through Relation Expansion, Upper-Level Ontologies and Automatic Reasoning

**DOI:** 10.1371/journal.pone.0022006

**Published:** 2011-07-18

**Authors:** Robert Hoehndorf, Michel Dumontier, Anika Oellrich, Dietrich Rebholz-Schuhmann, Paul N. Schofield, Georgios V. Gkoutos

**Affiliations:** 1 Department of Genetics, University of Cambridge, Cambridge, United Kingdom; 2 Department of Biology, Institute of Biochemistry and School of Computer Science, Carleton University, Ottawa, Ontario, Canada; 3 European Bioinformatics Institute, Wellcome Trust Genome Campus, Hinxton, Cambridge, United Kingdom; 4 Department of Physiology, Development and Neuroscience, University of Cambridge, Cambridge, United Kingdom; 5 The Jackson Laboratory, Bar Harbor, Maine, United States of America; Rutgers University, United States of America

## Abstract

Researchers design ontologies as a means to accurately annotate and integrate experimental data across heterogeneous and disparate data- and knowledge bases. Formal ontologies make the semantics of terms and relations explicit such that automated reasoning can be used to verify the consistency of knowledge. However, many biomedical ontologies do not sufficiently formalize the semantics of their relations and are therefore limited with respect to automated reasoning for large scale data integration and knowledge discovery. We describe a method to improve automated reasoning over biomedical ontologies and identify several thousand contradictory class definitions. Our approach aligns terms in biomedical ontologies with foundational classes in a top-level ontology and formalizes composite relations as class expressions. We describe the semi-automated repair of contradictions and demonstrate expressive queries over interoperable ontologies. Our work forms an important cornerstone for data integration, automatic inference and knowledge discovery based on formal representations of knowledge. Our results and analysis software are available at http://bioonto.de/pmwiki.php/Main/ReasonableOntologies.

## Introduction

Understanding the meaning of data is essential for accurate scientific analysis and interpretation. Ontologies formalize the meaning of terms in a vocabulary and provide a mechanism to integrate knowledge from different sources through semantic annotation of data. Interoperability of ontological resources is required to automatically analyze data across different data repositories and to enable automatic reasoning for knowledge discovery. One milestone has been the development and establishment of ontologies in the biomedical research community with the goal of integrating knowledge from different scientific resources and domains. In recent years, more emphasis has been put on the standardization, formalization and interoperability of the data resources and ontologies that characterize them [1]. However, the proliferation of species- and domain-specific ontologies has resulted in an urgent need to develop an approach to bridging the increasing gaps between these ontologies. It has now become necessary to automatically resolve inconsistencies across these resources to facilitate automated reasoning, formulation of complex queries across a variety of data resources, testing of hypotheses against the current body of knowledge and translational research [2].

Automated reasoning is the process of inferring automatically information from an ontology that is not directly asserted but implied by the axioms and definitions in the ontology. Substantial progress has been made in enabling reasoning over part-whole relations in biomedical ontologies [3], [4] and using automated reasoning over domain-specific upper-level ontologies to integrate ontologies of different domains [5], [6]. Automated reasoning has further been applied to classify proteins [7], to verify and complete the asserted axioms in biomedical ontologies [8], [9] and to identify relations between phenotype and disease [10]. Despite significant progress towards enabling automated reasoning over biomedical ontologies, large-scale automated reasoning is often limited by the size and complexity of the ontologies [11].

Questions of the type “Which genes are involved in abnormalities of the vertebrate vascular system localized within abdominal organs?” require in-depth knowledge of gene structure, taxonomy, anatomy, development and disease. To answer this question automatically, knowledge must be encoded in such a way that it becomes accessible to machines and allows the integration of the increasing amounts of data encoded using a variety of data formats and stored across numerous unconnected databases.

Biomedical ontologies, including the Gene Ontology (GO) [12], the Mammalian Phenotype Ontology (MP) [13] and the Human Phenotype Ontology (HPO) [14], offer a set of terms and descriptions in their domains, and considerable effort and resources have been devoted to their construction [15]. In order to realize their potential, ontologies must provide rich, explicit and consistent descriptions, so that automated systems are able to process and distinguish the meaning of their terms and use them to infer new information. Such descriptions are currently being created for ontologies within the biomedical domain. In particular, formal class definitions describe a class in terms of logical combinations of other classes and relations. In contrast to informal descriptions of classes, formal class definitions can be utilized for automated reasoning. Formal definitions of classes in GO, MP and HPO were recently introduced using a combination of manual and automated methods [8], [9], [16]. Because these formal definitions are based on classes and relations from several ontologies, they are called “cross-products”, and the cross-product definitions for GO, MP and HPO are called GO-XP, MP-XP and HPO-XP, respectively.

However, these definitions do not always make their semantics sufficiently explicit and accessible to automated reasoning, which limits their ability to inter-operate with ontologies of other domains and to facilitate knowledge discovery. In particular, many biomedical ontologies are represented in the OBO Flatfile Format for which the specification of an explicit semantics is currently work in progress [17]–[19]. Here, we demonstrate how to utilize biomedical ontologies in a formal representation based on the Web Ontology Language (OWL) [20] and use this formal representation for automated reasoning, consistency verification and knowledge discovery. To achieve this goal, we extend a method for formalizing biomedical ontologies using OWL [18], develop and apply an upper-level ontology [21] and derive an ontology of relations from those used in biomedical ontologies. We apply this method to the GO-XP, MP-XP and HPO-XP cross-product definitions to identify *unsatisfiable* classes. An *unsatisfiable* class is a class that could not possibly have any instances due to a contradiction in the axioms and definitions that restrict the class. The presence of an unsatisfiable class in an ontology is an indication of a mistake either in the structure of the ontology or the formal definition of the class. The consistent formulation of class definitions is necessary to utilize biomedical ontologies for answering powerful, cross-ontology queries and discovering new knowledge. Here, we demonstrate how to remove contradictory definitions and utilize the ontologies for expressive queries based on reasoning over ontologies.

## Materials and Methods

### Formal ontology

An ontology is a conceptualization of a domain of knowledge [22] and is used to make the meaning of terms in a vocabulary explicit and amenable to automated processing [23]. Ontologies contain classes which are arranged in a taxonomy and restricted through axioms. Examples of classes are *Bone*, *Apoptosis*, *Process* or *Hypoplasia*. Classes can have instances [24]. For example, a particular bone is an instance of *Bone* and a particular apoptosis process occurring in one cell at a particular time is an instance of *Apoptosis*. When classes in an ontology stand in an **is-a** relation, every instance of one class is also an instance of the other class [25]. The class *Apoptosis* and the class *Process* can stand in such a relation: every instance of *Apoptosis* is an instance of *Process*. Furthermore, classes can be restricted through axioms [26]. For example, *Apoptosis* can be restricted by an axiom that requires every instance of *Apoptosis* to have an instance of *Cell* as a participant.

### Upper level ontology

Ontologies from different domains may be integrated by alignment to an upper level ontology. An upper-level ontology provides a common foundation for classes and relations [21]. Typical classes found in upper-level ontologies include *Process*, *Material object*, *Quality* and *Function*. Upper-level ontologies further provide relations that can hold between *instances* of their classes. Commonly included relations are **has-part**, **has-participant** and **quality-of**. Several upper-level ontologies are well established including the Basic Formal Ontology (BFO) [27], the Descriptive Ontology for Cognitive and Linguistic Engineering (DOLCE) [28] and the General Formal Ontology (GFO) [24].

**Figure 1:**
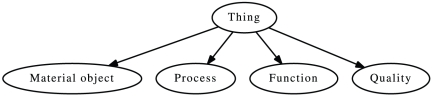
Taxonomy of the upper-level ontology. The four classes *Material object*, *Process*, *Quality* and *Function* are mutually disjoint.

For the purpose of this study, and to maximize compatibility with different upper-level ontologies, we use a fragment of these ontologies that consists of only four classes: *Material object*, *Process*, *Quality* and *Function*. We declare these four classes as mutually disjoint. The instances of *Material object* exist with all their parts at a time point and need no other entity to exist. Processes, on the other hand, are temporally extended and cannot exist at a single time point. Functions are capabilities or potentials for the occurrence of processes [29] and depend on material objects. We treat qualities as attributes of other entities. In BFO, qualities can only be attributes of independent continuants (*Material object* in our upper-level ontology), while both GFO and DOLCE allow qualities of material objects, processes and functions. In MP-XP and HPO-XP, qualities are frequently applied to functions and processes, and therefore we take the more liberal approach and do not restrict the kind of entities which qualities characterize. Figure 1 shows the taxonomy of this basic ontology and Table 1 shows the relations we include in our ontology.

**Table 1:** Relations in our upper-level ontology.

Relation	Domain	Range	Inverse relation
**function-of**	*Function*	*Material object*	**has-function**
**inheres-in**	*Quality*	*Thing*	**has-quality**
**derives-from**	*Material object*	*Material object*	
**has-participant**	*Process*	*Material object*	**participates-in**
**has-input**	*Process*	*Material object*	**input-of**
**has-output**	*Process*	*Material object*	**output-of**
**has-central-participant**	*Process*	*Material object*	**central-participant-of**
**part-of**	*Thing*	*Thing*	**has-part**
**proper-part-of**	*Thing*	*Thing*	**has-proper-part**
**realized-by**	*Function*	*Process*	**realizes**
**results-in**	*Process*	*Process*	

Relations in our upper-level ontology, implemented as OWL object properties, along with their domain and range restrictions, super-relations and their inverse relations.

The first step in our method creates a foundation of the domain classes in this upper-level ontology. In the class definitions of the ontologies we consider, PATO [30], the Foundational Model of Anatomy (FMA) [31], the Adult Mouse Anatomy Ontology (MA) [32], the Cell type Ontology (CL) [33], the Protein Ontology (PRO) [34], the Mouse Pathology Ontology [35], the ChEBI ontology of chemical structures [36], the UBERON cross-species anatomy ontology [8] and the Gene Ontology (GO) [12] are used. We assume that all classes in PATO are subclasses of *Quality*, while FMA, MA, CL, PRO, ChEBI and the Cellular Component branch of GO contain subclasses of *Material object*. We assume that the biological process branch of GO contains subclasses of *Process*. The *Molecular function* branch of GO may contain subclasses of either *Function* or *Process*, a problem of which the GO curators are aware [37]. Consequently, we performed our analysis twice using both assumptions.

### Relations in biomedical ontologies

Based on the upper-level ontology, we introduce a set of relations that hold between the *instances* of the classes in our ontology. We base our selection of relations on those that are used in biomedical ontologies, such as those listed in Table 2. Each relation in our ontology includes basic axioms pertaining to reflexivity, transitivity and symmetry. In addition, each relation determines the kinds of entities between which it is asserted (domain and range restrictions). This ensures that employing a relation in an axiom has consequences that can be inferred using an automated reasoner. In particular, it allows for the automated detection of inconsistencies and contradictory definitions such as those arising from modelling errors. Table 1 shows the relations we include together with their domain and range restrictions. A similar assignment of domain and range restrictions for common relations in biomedical ontologies can be found in *bridging* ontologies available from the OBO Foundry [1]. These bridging ontologies use classes from BFO [27] to restrict the domain and range of relations. The axioms we include for the relations are compatible with the axioms for relations in RO and BFO [38]. We are more liberal in our axioms for the **inheres-in** relation in that its range is *Thing*, because both MP-XP and HPO-XP use the **inheres-in** relation for material objects, processes and functions.

**Table 2:** 20 most frequent relations in OBO.

Relation name	Number of times used in ontologies	Number of times used in formal definitions
located_in	294126	58
part_of	97113	6383
partial_overlaps	39381	0
regional_part_of	39329	0
constitutional_part_of	24808	0
tributary_of	18428	0
branch_of	14351	0
regulates	8586	3654
hasMapping	7263	0
negatively_regulates	7063	2980
positively_regulates	6966	2946
has_rank	6028	0
sequence_of	5906	0
systemic_part_of	5340	0
develops_from	5129	175
start	5019	0
end	5011	0
has_functional_parent	4363	0
has_role	4145	0
surrounded_by	2444	2

The first column states the name of the relation, the second how often the relation is used in any OBO ontology, while the final column indicates occurrence of the relation in formal definitions. We performed the analysis on the full OBO library of ontologies (as of Nov 25, 2010), excluding the NCI Thesaurus, the BFO, the RO, the mappings of OBO ontologies to other ontologies and databases and the logical definitions of OBO ontologies. The full list is available on our project website.

Relations in the Open Biomedical Ontologies [1] are commonly asserted between classes [38]. For example, *Nucleus part-of Cell* is a statement involving the classes *Nucleus* and *Cell* and the *part-of* relation between classes. These relations between classes are then defined using another relation between instances according to a template provided by the OBO Relationship Ontology (RO) [38]. To illustrate the distinction between relations that hold between classes and relations that hold between individuals, we use *italic font* for relations between classes and **bold font** for instance-level relations. We will further call relations between classes *CC-relations* (for class-class) within this section to distinguish them from OWL relations between instances.

To use template definitions for CC-relations in class definitions, we must extend the method of defining CC-relations provided by RO to accommodate the possibility of their application in class intersections and unions [18]. For this purpose, we treat CC-relations in biomedical ontologies as templates that characterize a class based on a *single* argument. For example, we treat the relation *part-of* as a template which requires a single class as argument and represents the description of a class. “*part-of Cell*” then becomes a description of the class “**part-of** some *Cell*”, i.e., the class of things that are part of a cell. The statement “*Nucleus part-of Cell*” will then be an assertion that the class *Nucleus* is a subclass of “**part-of** some *Cell*”. To formalize and implement this approach to defining CC-relations, we modify the OWLDEF method and software [18].

OWLDEF provides a means to convert ontologies from the OBO Flatfile Format [17] into OWL [20] while expanding CC-relations according to the definitions provided by the RO [38]. While OWLDEF follows the RO approach in that CC-relations expand to *class axioms* in OWL (either a subclass, equivalent class or disjointness axiom), we modified this approach to expand CC-relations to *class descriptions*. Instead of templates with two variables, as in the original OWLDEF approach, we use templates with a single variable. The advantage of this approach is that class descriptions can be used in conjunction with intersections or unions, while class axioms cannot [39]. Additionally, we can reproduce RO's relation definitions by assuming that the first argument of any relation will always be declared as a subclass of the class description that results from use of the class construction template. In general, the assertion of “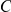 *R* 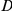
” is expanded to C SubClassOf: E where 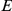 is the class resulting from expansion of *R* 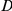 according to our method.

This method of defining CC-relations allows for their reuse and therefore enables the integration and interoperability of ontologies that employ the same or similar CC-relations. The deviation from the RO's method of defining CC-relations allows the application of this strategy to term definitions while maintaining compatibility with RO [18].

Before the consistency of a biomedical ontology can be verified with respect to the upper-level ontology, we must relate the relations (between individuals) used in a biomedical ontology to the relations in the upper-level ontology. This step is performed manually, based on an analysis of the meaning of relations in the biomedical ontology. For example, we assert that the relations labelled **has_part**, **has-part** and **has part** in ontologies we examined are equivalent.

As a next, optional step, axioms for domain classes can be added using the relations and classes available in the upper-level ontology. For example, PATO distinguishes between qualities of processes and qualities of physical entities [30]. After converting the examined ontologies to OWL and combining them with the upper-level ontology, we can add axioms to their classes explicitly to ensure that qualities of processes must inhere in processes, and qualities of physical objects must inhere in material objects.

The resulting ontology may be classified using an automated OWL reasoner such as Hermit [40], Fact++ [41] or Pellet [42]. Based on the resulting classification, we can perform queries, e.g., query for unsatisfiable classes or classes satisfying complex conditions.

### Using templates to repair ontologies

One common cause of contradictory class definitions is the ambiguous use of relations, i.e., with different meanings. Using relation definitions and OWL reasoning, we can disambiguate these different meanings. For example, the relation **has-central-participant** is a relation that holds between processes and material objects. However, it is also sometimes used as a relation between a quality and a material object, with the intended meaning that the quality inheres in a process that has a material object as participant.

To address this problem, we identify the different meanings in which a relation in an ontology is used, and provide a relation definition for each meaning. For example, the *has-central-participant* relation can have the meanings **has-central-participant** some ?Y and **inheres-in** some (**has-central-participant** some ?Y). Once all the possible ways in which a relation is used are formalized, we connect the resulting definitions disjunctively. In our example, the resulting statement would be:

has-central-participant some ?Y or

inheres-in some (has-central-participant some ?Y)

This statement is then used as the definition of *has-central-participant* in MP-XP. If *has-central-participant* is used as a relation between a process and a material object, the first part of the definition will become true (and the second false). If it is used as a relation between a quality and a material object, the second part of the definition becomes true (and the first false).

This method allows us to remove contradictions when a relation is used in a limited number of formally disjoint meanings. For this purpose, the application of these disambiguation templates require manual analysis and knowledge of the ontologies to which they are applied. We performed a manual evaluation of the use of disambiguation templates within HPO-XP and MP-XP, and found that all relations were correctly disambiguated through the use of these templates.

We may still be interested in identifying the particular class descriptions where a relation is used outside its intended meaning. With an appropriate query, OWL reasoning can provide an answer to this question. We defined ambiguous relations using a disjunctive statement. Because one part of the disjunction will always be unsatisfiable, the automated reasoner will eliminate this possibility and automatically infer that the only remaining option must apply. We can then query for the two distinct meanings of relations and obtain a list of results, which can then be added to the ontology's class definitions.

In MP-XP, we identified two relations with ambiguous use that lead to unsatisfiable class definitions. The first is *has-central-participant*, the second *inheres-in*. In the resulting OWL ontology, we use an OWL reasoner to perform a query for subclasses of:

inheres-in some (has-central-participant some Thing)

and obtain a list of 280 classes for which the second meaning in our disambiguation step is the only satisfiable option. We can now define a new class-level relation based on the template inheres-in some (has-central-participant some ?Y) and replace the wrongly asserted *inheres-in* or *has-central-participant* relations with this new relation.

### Reasoners and software

To perform our experiments, we used the Protege Ontology Editor [43] and the HermiT OWL reasoner (version 1.3.1) [40] on a dual core 3.20 GHz Intel Xeon CPU with 3 GB memory.

We developed a set of scripts and prototypical software libraries to prepare and analyze our data. The software and data we used, including the specific versions of the ontologies and their definitions, are available from our project website. The software includes

- a library to convert OBO files to OWL using the modified OWLDEF templates we developed,
- scripts to automatically assign super-classes from our upper-level ontology to classes in the used ontologies,
- scripts to count relations used both in formal definitions and relationship statements in OBO ontologies,
- a script to count the number of defined terms in an OBO ontology file.

The software we developed is written in Java and Groovy and depends on the OWLAPI [44].

### Ontology versions

Our analysis has been performed with the MP, HPO and GO as well as the formal definitions created for them. All ontologies and their definitions were obtained from the OBO Foundry website (http://obofoundry.org). The ontology files for the MP, HPO and GO were downloaded on November 25, 2010 and we have made a copy of the ontology files available on our project website.

## Results

**Figure 2:**
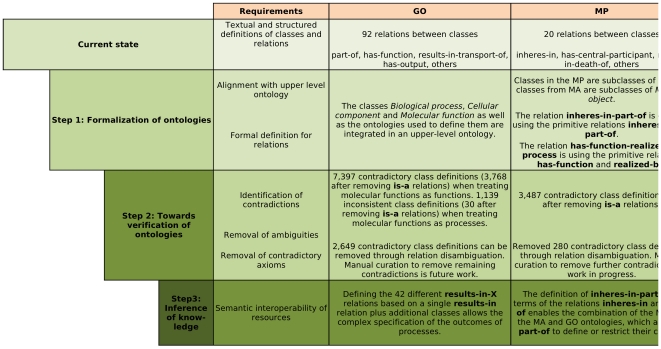
Overview of method and results.

The formal definitions of GO-XP, MP-XP and HPO-XP are work in progress [8], [9], as is most work on biomedical ontologies, since the definitions are subject to change and future revision [1]. A method to detect contradictory definitions can improve the quality of these definitions and improve the speed at which they are created. Our analysis was performed with a version of the GO definitions that contains 14,792 defined terms, while the HPO definitions contain 3,746 and the MP definitions 5,428 defined terms. Figure 2 provides an overview of our method and main results.

### Formalizing the semantics of ontologies and their relations

In GO-XP, the most frequently used relations are *part-of*, *has-output*, *has-input* and *regulates*. Slightly less than half of the relations are composite relations of the type *results-in*, i.e., those that are formed from the primitive **results-in** relation and classes. For instance, *results-in-binding-of* is composed of **results-in** and the class *Binding*. For some relations, we cannot yet identify appropriate process terms. For example, we could not identify an *increase in mass* process required to formalize *results-in-increase-in-mass-of*. Similarly, formalizing transport specific relations like *results-in-transport-from* or *results-in-transport-along* require a more fine-grained framework of transport processes. For the purpose of this study, we demonstrate the formalization using several examples (shown in Table 3), but do not expand any relation in GO-XP. We expand the relations *has-function-realized-by* and *inheres-in-part-of* in MP-XP and HPO-XP according to the templates in Table 3.

**Table 3:** Exemplary definition templates for relations used in biomedical ontologies.

Relation name	Definition template
*part-of*	**part-of** some ?Y
*inheres-in-part-of*	**inheres-in** some (**part-of** some ?Y)
*has-function-realized-by*	**has-function** some (**realized-by** only ?Y)
*capable-of*	**has-function** some (**realized-by** only ?Y)
*inheres-in-has-central-participant*	**inheres-in** some (**has-central-participant** some ?Y)
*has-input*	**has-input** some ?Y
*realized-by-has-input*	**realized-by** only (**has-input** some ?Y)

Each template unfolds into a class description in OWL and is represented using a modified form of the Manchester OWL Syntax.

Following this formalization of relations and classes, and using reasoning in the Web Ontology Language (OWL) [20], we identified 7,397 unsatisfiable classes in GO-XP under the assumption that the *Molecular function* branch of GO contains subclasses of *Function*. Assuming instead that the *Molecular function* branch of GO contains subclasses of *Process* allows us to identify 1,139 unsatisfiable classes. We identified 3,487 unsatisfiable class definitions in MP-XP and 1,017 in HPO-XP. Each unsatisfiable class definition indicates either an incorrect definition or a problem in the biomedical ontology for which the definitions were created.

To identify specific definitions that cause class unsatisfiability, we also performed our analysis after removing all explicitly asserted **is-a** relations from the ontologies. Under these conditions, we could identify 3,768 unsatisfiable classes in GO-XP when treating the *Molecular function* branch as subclasses of *Function* and 30 when treating the *Molecular function* branch as subclasses of *Process*. In MP-XP, we could identify 450 unsatisfiable class definitions and 245 in HPO-XP.

### Classification of contradictory class definitions

Among the identified contradictory class definitions, we can distinguish between local and global errors. Local contradictions arise from erroneous axioms within a single ontology. Global contradictions are the result of combining axioms from multiple ontologies. The unsatisfiable classes are identified using automated reasoning, and since we reason over these ontologies in an expressive formal language (OWL), we can identify many more formal problems whose resolution would be helpful to the developers of GO-XP [9], MP-XP and HPO-XP [8].

#### Local contradictions

**Figure 3:**
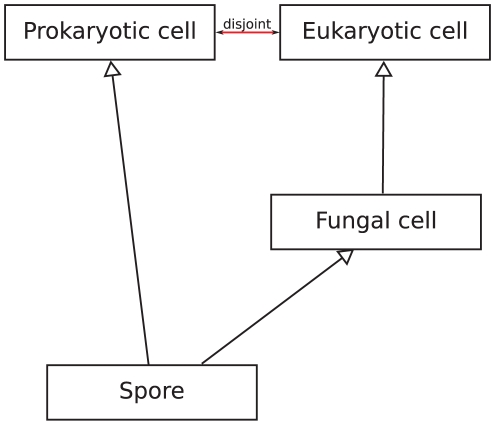
Local contradiction in the Cell type Ontology. The contradictory class definition arises from the assertion that *Spore* is both a *Prokaryotic cell* and a *Fungal cell*. *Fungal cell* is a kind of *Eukaryotic cell* which is disjoint from *Prokaryotic cell*.

The class *Spore wall assembly* (GO:0042244) illustrates a local contradiction that results from the contradictory definition of *Spore*. *Spore* is defined as the intersection of *Fungal cell* and *Prokaryotic cell*. *Fungal cell*, however, is a subclass of *Eukaryotic cell* which is disjoint from *Prokaryotic cell* leading to the unsatisfiability of *Spore* (see also Figure 3). From the unsatisfiability of *Spore* result the unsatisfiabilities of classes that use *Spore* in its definitions: *Sporulation resulting in formation of a cellular spore* is defined using *Spore*, *Spore wall biogenesis* is a part of *Sporulation resulting in formation of a cellular spore* and *Spore wall assembly* is a part of *Spore wall biogenesis*. All these classes are unsatisfiable due to the unsatisfiability of *Spore*. These unsatisfiable classes have been identified by an automated reasoner, leading to the conclusion that consistency verification of the ontologies and automated reasoning during ontology development can prevent such problems, as long as sufficiently expressive formal languages are used. We submitted the inconsistency of *Spore* to the developers of the Cell type ontology, and the underlying problem has been resolved in recent versions.

#### Global contradictions

Contradictory class definitions: Global contradictions results from contradictory definitions that arise from axioms constructed from multiple ontologies. One example of such an unsatisfiable class is *Leukocyte activation* (GO:0045321), which causes all of its subclasses to be unsatisfiable as well. *Leukocyte activation* is defined as a *Cell activation* that **has-input** some *Leukocyte*. In addition, *Leukocyte activation* is a subclass of *Cell activation* and *Immune system process*. Furthermore, *Cell activation* is a subclass of *Cellular process* while *Immune system process* is defined as a biological process which **has-agent** an *Immune system*. Therefore, through automated reasoning we find that *Immune system process* is a kind of *System process*: *System process* is defined as *Biological process* and **has-agent** some *Anatomical system* and *Immune system* is a subclass of *Anatomical system*. *System process*, in turn, is a subclass of *Multicellular organismal process* which is disjoint from *Cellular process*. Therefore, *Leukocyte activation* is unsatisfiable.

**Figure 4:**
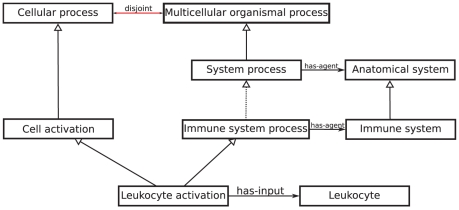
Global contradiction in the GO-XP. The contradiction arises from the inference that *Immune system process* is a kind of *System process*. *System process* is a kind of *Multicellular organismal process* which is disjoint from *Cellular process*.

Detecting this contradictory class definition relies on reasoning over the UBERON cross-species anatomy ontology [8] (to infer that *Immune system* is a type of *Anatomical system*) and reasoning over the formal definitions in GO (to infer that *Immune system process* is a *System process*). This is illustrated in Figure 4.

Another example of a global contradiction is found in MP-XP. The class *Liver inflammation*, a subclass of *Abnormal liver physiology*, is unsatisfiable. *Abnormal liver physiology* is defined as a *Functionality* that inheres in the *Liver*, while *Liver inflammation* is defined as an *Increased rate* that inheres in the *Inflammatory response* in which a *Liver* participates. *Inflammatory response* is a process from the GO, while *Liver* is a material object in MA. The **inheres-in** relation is functional, i.e., a quality can inhere in at most one thing. As a consequence, a quality that inheres both in *Inflammatory response* and *Liver* will be inferred to inhere in something that is both a liver and an inflammatory response at the same time. Since processes and material objects are disjoint, the resulting class is detected as unsatisfiable.

*Liver inflammation* is further defined as a subclass of *Abnormal liver physiology*, which is defined using the quality *Functionality* from PATO. According to PATO, *Functionality* must be a quality of a material object while *Increased rate* (used in defining *Liver inflammation*) must be a quality of a process. Therefore, another cause for the unsatisfiability of *Liver inflammation* is the definition of qualities in PATO. Removing only one cause for the unsatisfiability of *Liver inflammation* would therefore not remove the problem with *Liver inflammation*.

**Figure 5:**
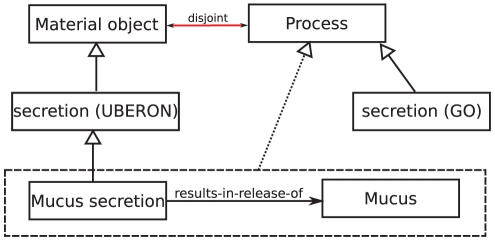
Contradiction in the GO-XP arising from faulty class definition due to homonymy. *Mucus secretion* is asserted to be a subclass of *Secretion*, an anatomical entity in the UBERON ontology which is a kind of *Material object*. Due to the domain and range restrictions of the relation **results-in-release-of**, *Mucus secretion* is inferred to become a kind of *Process*, which is disjoint from *Material object*. Use of the class *Secretion* from GO would have prevented this error.

**Contradictions through homonymy:** If two classes with different definitions share the same label, then the label of the two classes is called a homonym. Any homonym can be the cause of contradictions due to incorrect class assignments in the ontological framework founded in the polysemy of the homonym. One example of a contradictory class definition arising from homonymy is *Mucus secretion*. *Mucus secretion* is defined as the intersection of *Secretion* (UBERON:0000456) and **results-in-release-of** some *Mucus*. The class named *Secretion* in the UBERON ontology is a subclass of *Material object* while the GO class *Secretion* (GO:0046903) is a subclass of *Process*. Since the relation **results-in-release-of** (and any other **results-in** relation) must have a process as its first argument and *Process* and *Material object* are disjoint, we detect this contradiction automatically. Such contradictions can be the result of applying lexical methods to create formal definitions. Figure 5 illustrates this example.

**Contradictions from improper and ambiguous use of relations:** The following example shows an improper use of a well-defined relation. The class *Host cell cytoplasm part* (GO:0033655) is defined as *Host cell part* and **inheres-in** some *Cytoplasm*. The **inheres-in** relation is a relation between a *Quality* and an individual, resulting in *Host cell cytoplasm part* becoming a subclass of *Quality*. *Quality* and *Host cell part* (a subclass of *Material object*) are disjoint and therefore *Host cell cytoplasm part* is unsatisfiable.

A principal mistake in the MP-XP and HPO-XP is the use of the *has-central-participant* relation between qualities (instead of processes) and material objects, and a mistake in the GO-XP is the ambiguous use of relations between processes and functions (assuming that functions are disjoint from processes). For example, the relations *has-output* and *has-input* are frequently applied both to functions and processes and therefore a source of numerous unsatisfiable class definitions. Disambiguation templates can aid in the automatic detection and correction of ambiguous relations through a relaxation of a relation definition. The following disambiguation templates can be applied to *has-output*, *has-input* and *inheres-in*:

- *has-output* X: either the processes that have 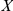 as output or the functions that are realized through processes that have 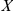 as output. For example, in the statement *has-output D-glucose*, the resulting class is either the class of processes that have D-glucose as output, or the class of functions that are realized through processes that have *D-glucose* as output.
- *has-input* X: either the processes that have 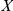 as input or the functions that are realized through processes that have 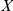 as input. For example, in the statement *has-input Lactose*, the resulting class is either the class of processes that have lactose as input, or the class of functions that are realized through processes that have *lactose* as input.
- *inheres-in* X: either the qualities that inhere in 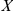 or the qualities that inhere in processes in which 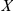 participates. For example, in the statement *inheres-in Liver*, the resulting class is either the class of qualities inhering in a liver, or the qualities that inhere in processes in which a liver participates.

Such templates allow the disambiguation of relations in biomedical ontologies. For example, in the GO-XP, relations such as *has-input* and *has-output*, which are applied to either processes or functions can explicitly be distinguished: either the relation is used in its intended meaning and the second part of the disjunction will become unsatisfiable, or it is applied to a function class that is realized by processes that satisfy the asserted condition, in which case the first part of the disjunctive relation definition is made unsatisfiable. Since one part of the disjunctive relation definition will always become unsatisfiable, we are able to disambiguate the relation through automated reasoning and query for the cases where the relation is used in its intended meaning (i.e., applied to a process class) and where it is used in its unintended meaning. Based on the results of such queries, we can then introduce new relations, e.g., *realized-by-has-input*, *realized-by-has-output* or *inheres-in-has-central-participant* (see Table 3).

We applied the templates for *has-input* and *has-output* to GO-XP, and the templates for *inheres-in* to MP-XP and HPO-XP, in each case after removing the asserted *is-a* relations. The application of the templates for *has-input* and *has-output* to the GO-XP removed 2,649 contradictory class definitions. Querying for uses of *has-input* in its intended meaning (applied to a process) yields 669 class definitions. On the other hand, when querying for the unintended meaning, expressed by the *realized-by-has-input* relation, results in 2,390 class definitions. The *has-output* relation is used in its intended meaning 462 times and in its unintended meaning (*realized-by-has-output*) 1,632 cases. In MP-XP and HPO-XP, we replaced both the *has-central-participant* and the *inheres-in* relations with the disambiguation templates. As a result, we removed 280 contradictions in MP-XP and 157 in HPO-XP.

We added the asserted *is-a* relations to MP-XP and HPO-XP after applying the disambiguation templates. In the MP-XP, 3,416 unsatisfiable classes remain and HPO-XP contains 1,016 unsatisfiable classes after applying the disambiguation templates to the ontologies including their *is-a* relations. This demonstrates that in most cases, multiple causes lead to classes becoming unsatisfiable in these ontologies.

### Knowledge integration and retrieval

The application of our method of formalizing the classes and relations in ontologies makes it not only possible to detect and repair some contradictions, but it can also be used to perform more expressive queries over the ontologies.

For example, we may be interested in finding phenotypes in mice that affect parts of the vascular system in any abdominal organ or its parts. We find, however, that no class in the MP can be retrieved, since none satisfies our query exactly. Although MP-XP defines a number of phenotypes that involve abnormalities in abdominal organs or the vascular system (such as liver abnormalities, kidney abnormalities or vascular abnormalities), no shared superclass ties these together. Our inference over both MP-XP and mouse anatomy allows us to infer that a number of phenotypes affect the vasculature of abdominal organs. Our retrieval yields 9 phenotypes: *Abnormal Kupffer cell morphology*, *Abnormal liver sinusoid morphology*, *Abnormal liver vasculature morphology*, *Abnormal renal plasma flow rate*, *Decreased renal plasma flow rate*, *Increased renal plasma flow rate*, *Enlarged liver sinusoidal spaces*, *Liver vascular congestion* and *Spleen vascular congestion*.

We obtain these results because our method expands the relation *inheres-in-part-of*. Without this expansion, inferences across both the phenotype and mouse anatomy ontologies would not be possible. The expansion of *inheres-in-part-of* using the primitive relations **part-of** and **inheres-in** enables inference over the parthood relations in the anatomy ontology. For example, *Liver vascular congestion* is defined both as an abnormality that *inheres-in-part-of Liver* and as an abnormality of a *Blood vessel*. After the expansion, *Liver vascular congestion* is defined as an abnormality of a *Blood vessel* which is **part-of** some *Liver*. Because, in the mouse anatomy ontology, a *Liver* is a kind of *Abdomen organ* and *Blood vessel* is a **part-of** the *Vascular system*, the definition of *Liver vascular congestion* satisfies our query.

In the analysis of the query results, we find that references to *Kidney* abnormalities are missing, although kidneys are abdominal organs as well. A manual inspection reveals that this is due to a missing assertion in the mouse anatomy ontology, i.e., that a *Kidney blood vessel* is a type of *Blood vessel*. The addition of this assertion enables us to retrieve kidney vascular abnormalities and has been requested from the curators of the mouse anatomy ontology.

## Discussion

The formalization of the meaning of terms in biomedical ontologies enables queries that can make reference to domain terminology in entirely new and unforeseen ways. These queries do not exclusively rely on specialized knowledge of the ontologies' structure and term names, but enable access to domain knowledge based on a term's meaning. Such a generalizable method is dependent on an upper level ontology that offers basic types and relations.

At the moment, biomedical ontologies often focus on including terms that are needed in different domains, adding natural language definitions to these terms, and connecting them using relations which are defined primarily in natural language. Consequently, understanding the meaning of these terms (and hence which inferences may be drawn from them) or performing queries that refer to them, requires extensive domain knowledge and a clear understanding of the structure of the ontologies in terms of their classes and relations.

While the need for domain expertise is not only desirable but essential, in the design of ontologies, modelling errors may not be avoided unless consistency verification through automated reasoning becomes a part of the ontology design process. The problem is even greater for ontologies or class definitions that are constructed automatically.

The application of our method shifts the focus of ontology development towards a *knowledge-based* perspective. From this point of view, the importance of natural-language definitions and explanations is matched by that of formalized and explicit semantics of terms and relations. Our method allows the explicit definition of the *meaning* of terms in more detail than before and therefore enriches their utility in automated processing and reasoning. The resulting definitions may then even be used to derive natural language definitions of relations and classes [45], ensuring consistency between both.

Through the application of our method, one goal of ontologies comes closer to realization: to improve knowledge discovery by providing a uniform method for relating and accessing data through formal semantics. The application of our method enhances the capacity of biomedical ontologies to achieve this goal. It benefits heavily from recent attempts to provide formal definitions of classes in biomedical ontologies by combining classes from multiple ontologies and expressive relations.

To provide a foundation for the classes and relations in biomedical ontologies, our method utilizes an upper-level ontology. To demonstrate the benefits gained through the use of such an ontology, we developed a minimal upper-level ontology that is applicable to the detection of mistakes and the inference of new cross-domain knowledge. This minimal ontology is a fragment of well-established ontologies such as the Basic Formal Ontology (BFO) [27], the Descriptive Ontology for Cognitive and Linguistic Engineering (DOLCE) [28], the General Formal Ontology (GFO) [24] or the Suggested Upper Merged Ontology (SUMO) [46]. Therefore, no, or only minimal, changes to our method are necessary when any of these ontologies is used as the upper-level ontology. In addition to providing compatibility with domain ontologies that are being developed using any established upper-level ontology, we can also derive a means to empirically evaluate upper-level ontologies based on how many incorrect class definitions can be automatically detected and subsequently repaired through their use.

### Conclusions

We provide a method for improving formal term and relation definitions in biomedical ontologies. Based on this method and through the use of automated reasoning, we have identified several thousand contradictory class definitions and could automatically repair some of them. These contradictions indicate either incorrect formal definitions or structural errors in the ontologies. The formalization method we propose improves the utility of automated reasoning over ontologies, so that it becomes possible to ask and answer more questions across multiple domains. We show that our motivating example of a query for all the genes in mice that are involved in abdominal vasculature abnormalities can be answered by applying our method. It is now possible to extend the range of queries by adding further connections through explicit relations between classes in ontologies. In particular, we can exploit links between a classification of species using the NCBI Taxonomy [47], and combine them with an ontology of species-independent anatomy (UBERON) [8] in order to retrieve a set of classes of vascular abnormalities in abdominal organs across all vertebrates. Furthermore, employing the Sequence Ontology (SO) [48] in the query will allow us to identify gene and protein sequences and their parts. These can then be related via the GO and the phenotype ontologies to the functions and processes that are involved in vascular abdominal abnormalities. All these ontologies, SO, UBERON, the phenotype ontologies and GO, are actively being developed to overcome the remaining barriers by adding new relations and connecting more domains. Provided that these ontologies focus on making their semantics explicit and their definitions and axioms consistent, as described by our method, more powerful questions will soon be answerable *through reasoning across ontologies alone*.
